# Replicative verification of susceptibility genes previously identified from families with segregating developmental dysplasia of the hip

**DOI:** 10.1186/s13052-021-01087-4

**Published:** 2021-06-26

**Authors:** Xiaowen Xu, Binbin Wang, Yufan Chen, Weizheng Zhou, Lianyong Li

**Affiliations:** 1 Department of Pediatric Orthopedics, Shengjing Hospital of China Medical University, Shenyang, 110004 PR China; 2 Center for Genetics, National Research Institute for Family Planning, 12 Dahuisi Road, Haidian District, Beijing, 100081 China

**Keywords:** Developmental dysplasia of the hip, Sanger sequencing, Sporadic sample verification

## Abstract

**Background:**

Developmental dysplasia of the hip (DDH) is a complex hip joint deformity with effects ranging from acetabulum malformation to irreversible hip dislocation. Previous studies suggest a significant association of four variations, teneurin transmembrane protein 3 (*TENM3*, OMIM * 610083) (chr4:183721398), heparan sulfate proteoglycan 2 (*HSPG2*, OMIM * 142461) (chr1:22201470), ATPase plasma membrane Ca^2+^ transporting 4 (*ATP2B4*, OMIM * 108732) (chr1:203682345), and prostaglandin F receptor (*PTGFR*, OMIM * 600563) (chr1:79002214), with DDH susceptibility in families with segregating DDH. However, the association was not validated in sporadic cases and remains controversial. To confirm the association of the reported variations in these four genes with DDH, we conducted replicative verification in 250 sporadic samples with DDH from a Chinese Han population.

**Methods:**

We conducted Sanger sequencing after amplifying the variation sites. The results were compared with the reference sequence from the GRCh37 assembly in UCSC (http://genome.ucsc.edu).

**Results:**

Replication analysis of 250 sporadic samples by Sanger sequencing indicated that the four variations, *TENM3* (OMIM * 610083, chr4:183721398), *HSPG2* (OMIM * 142461, chr1:22201470), *ATP2B4* (OMIM * 108732, chr1:203682345), and *PTGFR* (OMIM * 600563, chr1:79002214), were not associated with the susceptibility to DDH in the Chinese Han population.

**Conclusions:**

Further studies should be performed to identify other variations of these four genes that are potentially associated with DDH by whole-exome sequencing and the results should be verified in different populations.

## Background

Developmental dysplasia of the hip (DDH) is a common congenital malformation, which is related to hip osteoarthritis at an early age [1, 2]. DDH occurs at a rate of approximately 5 per 1000 live births [3]; diagnosis and therapy at an early stage are crucial for avoiding serious complications.

The etiology of DDH is multifactorial and includes both genetic and environmental factors [4]. Studies have shown that breech presentation, oligohydramnios, female gender, higher birth weight, and primiparity are environmental factors affecting DDH [4]. Monozygotic twins were shown to have a higher risk of DDH than dizygotic twins [5]. Additionally, people who have first-degree relatives with DDH are susceptible to a 12-fold higher risk of developing the condition [6], indicating the important role of genetic factors. However, the pathogenic genes causing DDH are yet to be identified [6]. Several genes, including WNT inhibitory factor 1 (*WIF1,* OMIM * 605186), Collagen type XI alpha 2 chain (*COL11A2,* OMIM * 120290), and Pregnancy-associated plasma protein A2 (*PAPPA2*, OMIM * 176385), were shown to increase susceptibility to DDH through candidate association studies in one population [7–10]. However, these results could not be confirmed in other populations.

DDH is a polygenic disease with complex genetic causes [10]. A series of studies on C-X3-C motif chemokine receptor 1 (*CX3CR1,* OMIM * 601470) provided methods for identifying DDH-related genes. Feldman et al. [11] found that the variant rs3732378 in *CX3CR1* causes a threonine to methionine amino acid change in the coding sequence. This variation was shared by four American DDH-affected family members, according to whole-exome sequencing linkage analysis. Li et al. [12] identified the variant rs3732378 in *CX3CR1* in a case-control study of thousands of sporadic DDH samples. Subsequent studies showed that ablation of *CX3CR1* affects acetabular morphology and gait in *CX3CR1* knock-out mice [11]. *PAPPA2* has also been shown to be related with DDH [9, 13].

Recently, several possible variations in *TENM3*, *ATP2B4*, *HSPG2*, and *PTGFR,* which are related to DDH, were detected by genome-wide linkage analysis of three families [5, 14, 15]. *TENM3* **(chr4:183721398)** was found to contain a novel A to C nucleotide alteration, causing a glutamine to proline change in a four-generation pedigree from Philadelphia, through linkage analysis [5]. Previous studies revealed two heterozygous rare coding variants in *HSPG2* (chr1:22201470) and *ATP2B4* (chr1:203682345) in a Saudi family with DDH and found that HSPG2 regulates ATP2B4 expression through transcription factors, as determined by *in silico* analysis [14]. A termination codon variation (c.922C>T) in *PTGFR* (chr1:79002214), which was located in exon 2/4 of chromosome 1, was detected in a Chinese family with DDH [15].

However, whether the variations *TENM3* (OMIM * 610083, chr4:183721398), *HSPG2* (OMIM * 142461, chr1:22201470), *ATP2B4* (OMIM * 108732, chr1:203682345), and *PTGFR* (OMIM * 600563, chr1:79002214) are related to DDH in sporadic samples remains unclear. Therefore, this study was conducted to identify the association between these four variations and DDH in a large sample of sporadic cases with DDH in a Chinese Han population.

## Methods

### Subjects

A total of 250 patients with sporadic DDH from a Chinese Han population participated in this study. All subjects were diagnosed at the authors’ institution. The patients were diagnosed with unilateral or bilateral DDH by two experienced pediatric orthopedics experts according to their medical history and based on the results of physical examination and pelvic radiograph. Patients with syndromic or neuromuscular hip dislocation were excluded. This study was approved by the Medical Ethic Committee of the authors’ institution. Informed written consent was obtained from the parents or guardians of all patients.

### Sanger sequencing

Blood samples were collected for genomic DNA extraction using the QIAamp DNA Blood kit (QIAGEN, Hilden, Germany) according to the manufacturer’s protocol. The concentration and quality of DNA were assessed with a Nanodrop spectrophotometer (Thermo, Delaware, USA). Variations in *TENM3*, *ATP2B4*, *HSPG2*, and *PTGFR* were obtained from a previous family linkage analysis [5, 14, 15] (Table 1). Primers for polymerase chain reaction (PCR) were designed using Primer 5 software and synthesized by Sangon Biotech (Shanghai, China) (Table 1). The reaction system was added according to the instructions of KOD-Multi & Epi kit (TOYOBO, Osaka, Japan) for *TENM3*. The amplification conditions were as follows: 94 °C for 2 min for pre-denaturation, then 98 °C for 10 s, 68 °C for 15 s, and 68 °C for 2 min for 40 cycles. And the amplification conditions used for *ATP2B4*, *HSPG2*, and *PTGFR* were 95 °C for 5 min for pre-denaturation, then 94 °C for 30 s, 58 °C for 30 s, and 72 °C for 60 s for 38 cycles, and followed by 72 °C for 10 min. The products of Sanger sequencing were analyzed using an ABI 3730XL Genetic Analyzer (Applied Biosystems, Foster City, USA). The results were compared with the reference sequence derived from GRCh37 assembly of UCSC (http://genome.ucsc.edu).

**Table 1:** Primer sequences

Gene	Variation location	Forward Primer	Reverse Primer
*TENM3*	chr4:183721398	5′-GCTCAACAACGCCTTCTACC-3′	5′-TGTGGGTCGTAACAGCAGTC-3′
*HSPG2*	chr1:22201470	5′-GGAAGGGTGATGGTTTGGAG-3′	5′-CAGAAAGATGGCAGTGGGAG-3′
*ATP2B4*	chr1:203682345	5′-AAAGCAGGGCCTGAATGGT-3′	5′-GAGGTTGCAGTGAGCTGAGAT-3′
*PTGFR*	chr1:79002214	5′-GTCTCCATCAGTGTTTCTGCTATC-3′	5′-GAATTTTCCAGTTAGGCTACAGGTA-3′

## Results

The four variations previously identified by linkage analysis of different families with DDH were not detected by Sanger sequencing of the 250 sporadic samples. These results indicate the variations in the pedigree segregating DDH in *TENM3* (OMIM * 610083, chr4:183721398), *HSPG2* (OMIM * 142461, chr1:22201470), *ATP2B4* (OMIM * 108732, chr1:203682345), and *PTGFR* (OMIM * 600563, chr1:79002214) are not associated with susceptibility to DDH in the Chinese Han population.

## Discussion

Previous studies showed that DDH is a polygenic malformation disease [4, 16]. And most of the DDH gene studies are candidate gene association studies at present, but family linkage analysis can find main effect genes of DDH. Linkage analysis studies can detect variations that are potentially related to DDH through pedigree analysis, although the results require confirmation through a series of experiments using different populations and animal models. Feldman et al [11] found that *CX3CR1* co-segregated with DDH families by linkage analysis and Li et al [12] verified the pathogenicity of the gene in sporadic cases subsequently, which identified a pathogenic gene systematically. The possible pathogenic genes currently identified by family linkage analysis are *TENM3, HSPG2, ATP2B4* and *PTGFR* gene, but none of them have been validated in the sporadic DDH.

Teneurin 3 belongs to a highly conserved family of proteins and is necessary for various functions, including cell adhesion, cytoskeleton interaction, and calcium binding [17, 18]. Previous studies revealed a novel mutation in *TENM3* (OMIM * 610083) *,* which co-segregated in all severely affected members in a three-generation family from Philadelphia. This mutation also delayed development of the left acetabulum and left glenoid fossa, as observed by Alcian blue staining in 8-week-old knock-in mutant mice [5]. Feldeman et al found that Matrix metallopeptidase 13 (*MMP13*, OMIM * 600108), which is related to chondrogenesis [19], was overexpressed in femur-derived bone cells of knock-in mice [5]. Previous studies showed that *TENM3* is related to cartilage formation and expressed in prechondrogenic mesenchymal cells.

*HSPG2* (OMIM * 142461) encodes the perlecan protein, a heparan sulfate proteoglycan [20], which localizes in basement membranes, vascular structures, cartilage, and osteogenic tissues [21] and participates in cellular proliferation, differentiation, and migration [22]. *In vitro* studies showed that perlecan regulates chondrocyte differentiation, which plays an important role in cartilage development [23]. Previous studies showed that Safranin-O staining of the cartilage and expression of the typical collagen network were decreased in perlecan-deficient mice [21], which may have affected the occurrence of DDH. Therefore, *HSPG2* may cause DDH by affecting cartilage growth.

Heterozygous variation in *ATP2B4* (OMIM * 108732) was observed in a Saudi family pedigree, with *HSPG2* shared by all three affected individuals in the family, as revealed by whole exome sequencing [14]. Using *in silico* analysis, Basit et al found that *ATP2B4* expression was regulated by *HSPG2* and involved various transcription factors [15]. Previous studies showed that ATP2B4 participated in the regulation of bone homeostasis through calcium signaling [24]. The expression level of *ATP2B4* was increased during cellular senescence in human mesenchymal stem cells; these cells show the potential to differentiate into chondrocytes, which are components of the hip [25, 26]. Thus, *ATP2B4* may play an important role in hip joint formation.

*PTGFR* (OMIM * 600563) , also known as FP, belongs to the G protein-coupled receptor family of seven transmembrane-spanning receptors. *PTGFR* plays an important role in chondrocyte differentiation and cartilage development [27–29]. PGF2α exerts its biological activities by binding to its receptor, *PTGFR* [30]. Previous studies showed that PGF2α stimulated the expression of cartilage marker genes in a rat cell line and human articular chondrocytes [30, 31]. Studies also revealed that *PTGFR* participates in chondrocyte hypertrophy differentiation by regulating Bmp signaling [28]. Therefore, *PTGFR* may affect hip joint development as a receptor of PGF2α, which promotes cartilage formation.

In this study, we did not observe an association between these four variations and DDH; however, the association between other sites in these four genes and DDH remains unclear. The 250 sporadic cases evaluated in this study were from a Chinese Han population, indicating the possibility of a population-specific result. The relationship between these variations and DDH in other populations requires further analysis.

## Conclusions

Our replication study indicated that no association exists between the four previously reported variations in *TENM3* (OMIM * 610083, chr4:183721398), *HSPG2* (OMIM * 142461, chr1:22201470), *ATP2B4* (OMIM * 108732, chr1:203682345), and *PTGFR* (OMIM * 600563, chr1:79002214) and DDH in a Chinese Han population. However, other sites in these genes were not evaluated. Further studies are needed to identify other variations in these genes that are potentially associated with DDH by whole-exome sequencing, followed by verification of the associations in different populations.

## Data Availability

The datasets used and analyzed in this present study are available from the corresponding author on reasonable request.

## List of abbreviations

DDH: Developmental dysplasia of the hip
TENM3: Teneurin transmembrane protein 3
HSPG2: Heparan sulfate proteoglycan 2
ATP2B4: ATPase plasma membrane Ca^2+^ transporting 4
PTGFR: Prostaglandin F receptor
WES: Whole-exome sequencing
WIF1: WNT inhibitory factor 1
COL11A2: Collagen type XI alpha 2 chain
CX3CR1: C-X3-C motif chemokine receptor 1
PAPPA2: Pregnancy-associated plasma protein A2
PCR: Polymerase chain reaction
GDF5: Growth differentiation factor 5
MMP13: Matrix metallopeptidase 13
